# Patients’ choices regarding online access to laboratory, radiology and pathology test results on a hospital patient portal

**DOI:** 10.1371/journal.pone.0280768

**Published:** 2023-02-03

**Authors:** Pauline Hulter, Wesley Langendoen, Bettine Pluut, Guus G. Schoonman, Remco Luijten, Femke van Wetten, Kees Ahaus, Anne Marie Weggelaar-Jansen

**Affiliations:** 1 Erasmus School of Health Policy & Management, Erasmus University, Rotterdam, The Netherlands; 2 Department of Neurology, Elisabeth-TweeSteden Hospital, Tilburg, The Netherlands; 3 Tilburg School of Humanities and Digital Sciences, Tilburg University, Tilburg, Netherlands; 4 Department of Rheumatology, Elisabeth-TweeSteden Hospital, Tilburg, The Netherlands; 5 ETZ Digital, Elisabeth-TweeSteden Hospital, Tilburg, The Netherlands; 6 D&A Medical Group B.V., Waardenburg, The Netherlands; 7 Clinical Informatics, Eindhoven University of Technology, Eindhoven, The Netherlands; Universitat Luzern, SWITZERLAND

## Abstract

The disclosure of online test results (i.e., laboratory, radiology and pathology results) on patient portals can vary from immediate disclosure (in real-time) via a delay of up to 28 days to non-disclosure. Although a few studies explored patient opinions regarding test results release, we have no insight into actual patients’ preferences. To address this, we allowed patients to register their choices on a hospital patient portal. Our research question was: When do patients want their test results to be disclosed on the patient portal and what are the reasons for these choices? We used a mixed methods sequential explanatory design that included 1) patient choices on preferred time delay to test result disclosure on the patient portal for different medical specialties (N = 4592) and 2) semi-structured interviews with patients who changed their mind on their initial choice (N = 7). For laboratory (blood and urine) results, 3530 (76.9%) patients chose a delay of 1 day and 912 (19.9%) patients chose a delay of 7 days. For radiology and pathology results 4352 (94.8%) patients chose a delay of 7 days. 43 patients changed their mind about when they wanted to receive their results. By interviewing seven patients (16%) from this group we learned that some participants did not remember why they made changes. Four participants wanted a shorter delay to achieve transparency in health-related information and communication; to have time to process bad results; for reassurance; to prepare for a medical consultation; monitoring and acting on deviating results to prevent worsening of their disease; and to share results with their general practitioner. Three participants extended their chosen delay to avoid the disappointment about the content and anxiety of receiving incomprehensible information. Our study indicates that most patients prefer transparency in health-related information and want their test results to be disclosed as soon as possible.

## Introduction

Patient portals are *“provider-tethered applications that allow patients to access*, *but not to control*, *certain healthcare information (e*.*g*., *their EHR [electronic health record]) and provide communication and administrative functions (e*.*g*., *secure messaging*, *appointment booking*, *and prescription refill requests)”* (p.2) [1]. Patients may or may not want to access their healthcare information, such as test results, on patient portals. Research has shown that patients’ reasons for looking at online test results are 1) more transparent health-related information [2,3], 2) being able to prepare their consultation with a healthcare professional [1,4,5], or 3) reduce their anxiety [5]. Or they may wish to avoid accessing their test results online if they are anxious about the results [5] or if they are concerned about not understanding the results [6]. Despite a few studies on the reasons for accessing and not accessing healthcare information online, patients’ preferences regarding when their results are disclosed on patient portals remain unknown [7].

Different healthcare providers disclose test results on patient portals at different time points. Patients may have immediate access to their results or they may need to wait several days. Published time delays for laboratory, radiology, and pathology test results range from immediate disclosure [8–10] to a four-day wait [11]. Some studies have reported immediate disclosure [12] or an unknown delay until disclosure [2] of laboratory results while other studies have reported time delays ranging from one to 14 days for the disclosure of radiology results [13,14]. In another study three working days after the result was finalized was mentioned [15].

Patients’ actual preferences on when their test results should be disclosed on patient portals have not been studied yet. This is interesting as these preferences are likely to differ. Victoor et al [16] stressed “*There is no such thing as the typical patient*: *different patients make different choices in different situations*.*”* (p.13). While we are aware of the different time delays for disclosure of healthcare information on patient portals, we still do not know what the actual patient choices are and why [17]. Our research question was: When do patients want their test results to be disclosed on the patient portal and what are the reasons for these choices?

## Materials and methods

### Design and study setting

We used a sequential explanatory mixed methods approach [18] to understand patients’ choices per specialty on when their laboratory, radiology and pathology results should be disclosed on a patient portal. The first part of our study was a quantitative study of data from a patient portal that included information on when patients wanted to receive their test results. The second part consisted of a qualitative study including seven semi-structured interviews of patients who changed their choices through the patient portal.

We divided the specialties into the following three categories to provide a structured overview of our results: 1) surgical specialties, 2) medical specialties, and 3) obstetrics and gynecology [19,20]. These categories are described further in Table 1.

**Table 1:** Categorization of medical specialties.

Surgical specialties	Medical specialties	Obstetrics and gynecology
Anesthetics	Allergology	Obstetrics and gynecology
Ear, nose, throat^a^	Cardiology	
Neurosurgery	Dermatology	
Ophthalmology	Gastroenterology	
Orthopedics	Internal medicine	
Plastic surgery	Neurology	
Urology^b^	Pulmonary medicine	

^a^This specialty is not categorized by the OECD [19] and the WHO [20]. In the Netherlands, this specialty is surgical.

^b^We followed the categorization of the OECD [19] instead of the WHO [20]. In the Netherlands, this specialty is surgical.

We used the patient portal of a Dutch teaching hospital as a convenience sample (502,000 outpatient visits, 789 beds, 5,218 employees). The patient portal was launched in March 2018 and offers functions, including online access to medical files and test results, an overview of all hospital appointments, an e-consult service where messages can be sent to healthcare professionals, and a repeat prescription service. Patients can also use the portal to complete questionnaires, give permission for their medical file to be shared with third parties (e.g., their general practitioner), and can make, change, or cancel appointments.

Since October 2020, patients can use the portal to choose the delays for disclosure of laboratory test results (blood or urine sampling) or radiology and pathology results. Patients have the following choices: 1) see their results after one day, 2) see their results after seven days, 3) see their results after fourteen days, 4) see their results after twenty-one days, or 5) see their results after twenty-eight days, or 6) never see their test results. Radiology and pathology results are disclosed after seven days to enable healthcare professionals to discuss results with patients.

When a patient makes a choice, the portal shows a follow-up question: “You have entered X days, are you aware that you might see the results before you have spoken to your healthcare professional?” The portal also gives patients the opportunity to change their initial preferences for each test at any time.

#### 1) Quantitative patient portal usage data on patient choices

The quantitative data set comprised information on all patient portal users who made either one or more choices on the portal regarding their laboratory and/or radiology and pathology results between October 2020 and April 2021. The independent variables collected of all patient portal users were age, gender, and initial preference for each test result. The dependent variable was the number of times the patient changed their mind about their initial choice for blood and urine, radiology and/or pathology test results.

Continuous variables were expressed as means ± standard deviations, medians, and minimums and maximums. Categorical variables were expressed as numbers and percentages. One researcher (WL) performed statistical analyses and differences between groups were deemed significant at a significance level of p ≤ .05. For normally distributed data (parametric), numerical data were analyzed with an unpaired t-test and categorical data with a chi-squared test. Non-parametric data were analyzed using a Wilcoxon rank-sum test. We compared patients who did not change their initial choice with the group of patients who did change their initial choice. There were no differences found between the groups, so we used descriptive statistics to describe the patient portal users in all groups: 1) all patient portal users, 2) patients who did not change their initial choice, and 3) patients who changed their initial choice. All data were analyzed using R version 4.1.1 [21].

#### 2) Qualitative interview data of patients who changed their choices

One researcher from the hospital (GS) invited 43 purposively sampled patients who changed their initial choice for an interview. Patients were invited by a letter explaining the study combined with an informed consent form. A reminder by telephone or e-mail was send after two weeks. The participants were offered a €20 gift card to participate. Of the 43 invited participants who changed their initial choice, seven (16%; two females, five males) responded, including one participant who changed his preferences more than once. Three of these seven participants changed their preference on laboratory, radiology and pathology results, three on laboratory results only, and one on radiology and pathology results only.

One researcher (PH) conducted semi-structured interviews with these seven participants following a predefined topic list (see S1 Appendix). The interviews were conducted by telephone and took on average 32 minutes (range 24 to 48 minutes). The interviews were audio recorded and transcribed verbatim.

We generated codes using thematic data analysis [22,23]. First, one researcher (PH) became familiar with the data by reading the transcripts in depth and then open coded the transcripts. Next, two researchers (PH, AW) discussed the open codes until consensus was reached. Some codes clearly fitted together into a broader theme, so one researcher (PH) performed axial coding. For instance, the open code ‘losing weight when sugar level rises’ belongs to the broader theme ‘acting on test results’. Finally, three researchers (PH, AMWJ, and BP) reviewed and modified themes until there was consensus on themes among the researchers.

### Ethics statement

The Ethical Committee of Erasmus University approved our research proposal (21–027) and checked compliance to General Data Protection Regulation guidelines.

## Results

### Measurements

#### Descriptive analysis: Characteristics of patients

In total, 4592 patients (1643 males, 35.8% and 2949 females, 64.2%) participated by indicating when they wanted to get their test results. The mean age of male participants was 56.3±15.3 years (median 59 years; range 12–95 years) while the mean age of female participants was 49.5±16.0 years (median 46 years; range 12–92 years). The 50–59-years age category contained the most patients (986 patients, 21%). In total, 4592 patients registered 13,780 choices (including changed preferences).

There was no significant difference in the mean age of portal users who changed their initial preference (N = 43; 50.3±15.4 years) and the mean age of portal users who did not change their initial preference (N = 4549; 49.6±16.5 years) (*p* = .76). There was also no significant difference in the proportion of male patients between the group that changed their initial choice (40%; N = 17) and the group that did not (36%; N = 1638) (*p* = .23). Because age and gender distribution were not significantly different between the groups, we did not take these variables into account when inviting participants for interviews.

#### Patients (initial) preference choices

Patients made choices about test results from at least 16 specialties. To start, all patients (N = 4592) made an initial choice, regardless of the specialty. The characteristics of patients making each choice are detailed in Table 2. For laboratory (blood and urine) results, 3530 (76.9%) patients chose a delay of 1 day and 912 (19.9%) patients chose a delay of 7 days for their results, indicating that most patients preferred quick access to their test results. Only a few patients never wanted to see their test results (0.4%) or after 14 or more days (2.8%) (Table 2.1). For radiology and pathology test results, 4352 patients (94.8%) chose the shortest delay of 7 days and only a few patients never wanted to have their test results (0.7%) or have their results after 14 or more days (4.5%) (see Table 2.2).

**Table 2:** Details of initial test result choices (N = 4592).

2.1 Laboratory test results							
Choice	Ave. age (years)	Min. age (years)	Max. age (years)	Male	Female	Total	% of total
1 day	48.5	12	95	1200	2330	**3530**	**76.9**
7 days	53.8	12	88	384	528	**912**	**19.9**
14 days	56.4	17	86	30	39	**69**	**1.5**
21 days	59.6	28	76	3	11	**14**	**0.3**
28 days	44.1	20	75	21	26	**47**	**1.0**
Never	43.2	20	76	5	15	**20**	**0.4**
**2.2 Radiology and pathology test results**							
**Choice**	**Ave. age (years)**	**Min. age (years)**	**Max. age (years)**	**Male**	**Female**	**Total**	**% of total**
1 day	-	-	-	0	0	**0**	**0.0**
7 days	49.7	12	95	1157	2795	**4352**	**94.8**
14 days	49.8	17	86	46	92	**138**	**3.0**
21 days	54.2	28	75	3	11	**14**	**0.3**
28 days	44.9	20	81	25	31	**56**	**1.2**
Never	49.7	25	82	12	20	**32**	**0.7**

#### Characteristics of patients who changed their preferences

Patients were allowed to change their preference on when they received their laboratory, radiology and pathology results. In total, 37 patients (13 males; 24 females) made 60 changes to their initial preference for laboratory test results for at least 16 specialties (subdivided into surgical, medical, and obstetrics and gynecology) (see Table 3). Two male patients changed their preference twice, namely, for internal medicine (N = 1) and for orthopedics (N = 2). Regardless of type of specialty, the results are comparable.

**Table 3:** Number of changes to laboratory test result preferences per specialty.

Specialty category (total changes)	Total changes (% of total choices per specialty category)	Male	Female
Surgical (2156)	21 (1)	8	11
Medical (2615)	22 (0.8)	9	12
Obstetrics and gynecology (532)	5 (0.9)	1	4
Unknown (1376)	12 (0.9)	4	8
*Total*	*60*	* *	* *

**Fig 1:**
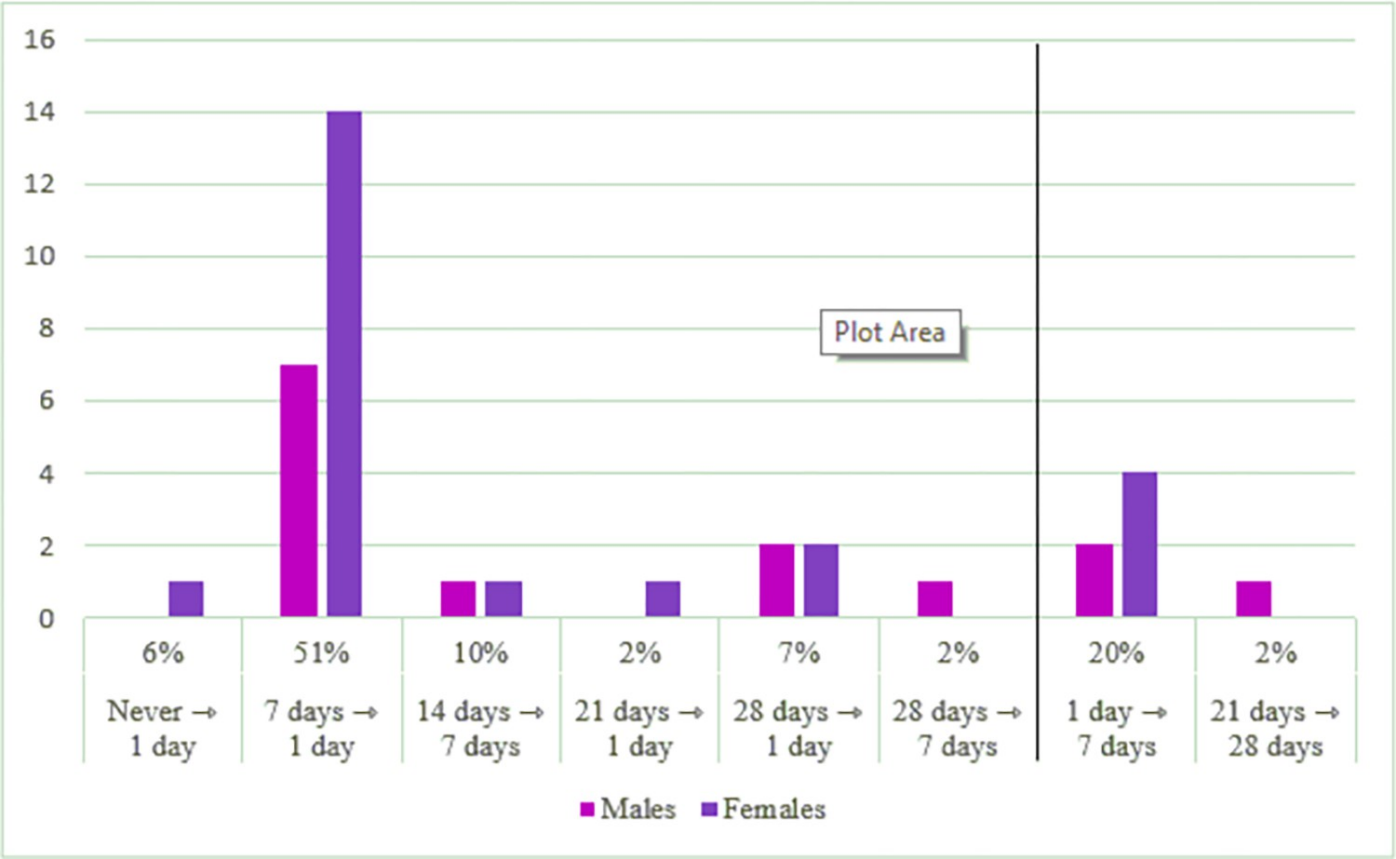
Preference changes for laboratory test results.

Of these changes, 47 (78%) were for a shorter delay, and 31 of these (51%) were from 7 days to 1 day, see Fig 1.

Concerning the time delay for getting radiology and pathology test results, 15 patients (10 males; 5 females) made 38 changes to their preference for at least 14 specialties (including ear, nose and throat, ophthalmology, orthopedics, plastic surgery, surgery, urology, allergology, cardiology, dermatology, gastroenterology, internal medicine, neurology, pulmonary medicine, and obstetrics and gynecology) (see Table 4). There were minimal differences in preference changes between specialties. One male patient changed his preference twice for cardiology (N = 1) and one female patient changed her preferences three times for dermatology (N = 1); ear, nose and throat (N = 1); gastroenterology (N = 1); and plastic surgery (N = 1). Strikingly, these two patients changed their preferences back to their initial preferences.

**Table 4:** Number of changes to radiology and pathology test result preferences per specialty category.

Specialty category (total changes)	Total changes (% of total choices per specialty category)	Male	Female
Surgical (1912)	14 (0.7)	6	4
Medical (2671)	20 (0.7)	6	9
Obstetrics and gynecology (532)	1 (0.2)	0	1
Unknown (1372)	3 (0.1)	1	1
*Total*	*38*	* *	* *

**Fig 2:**
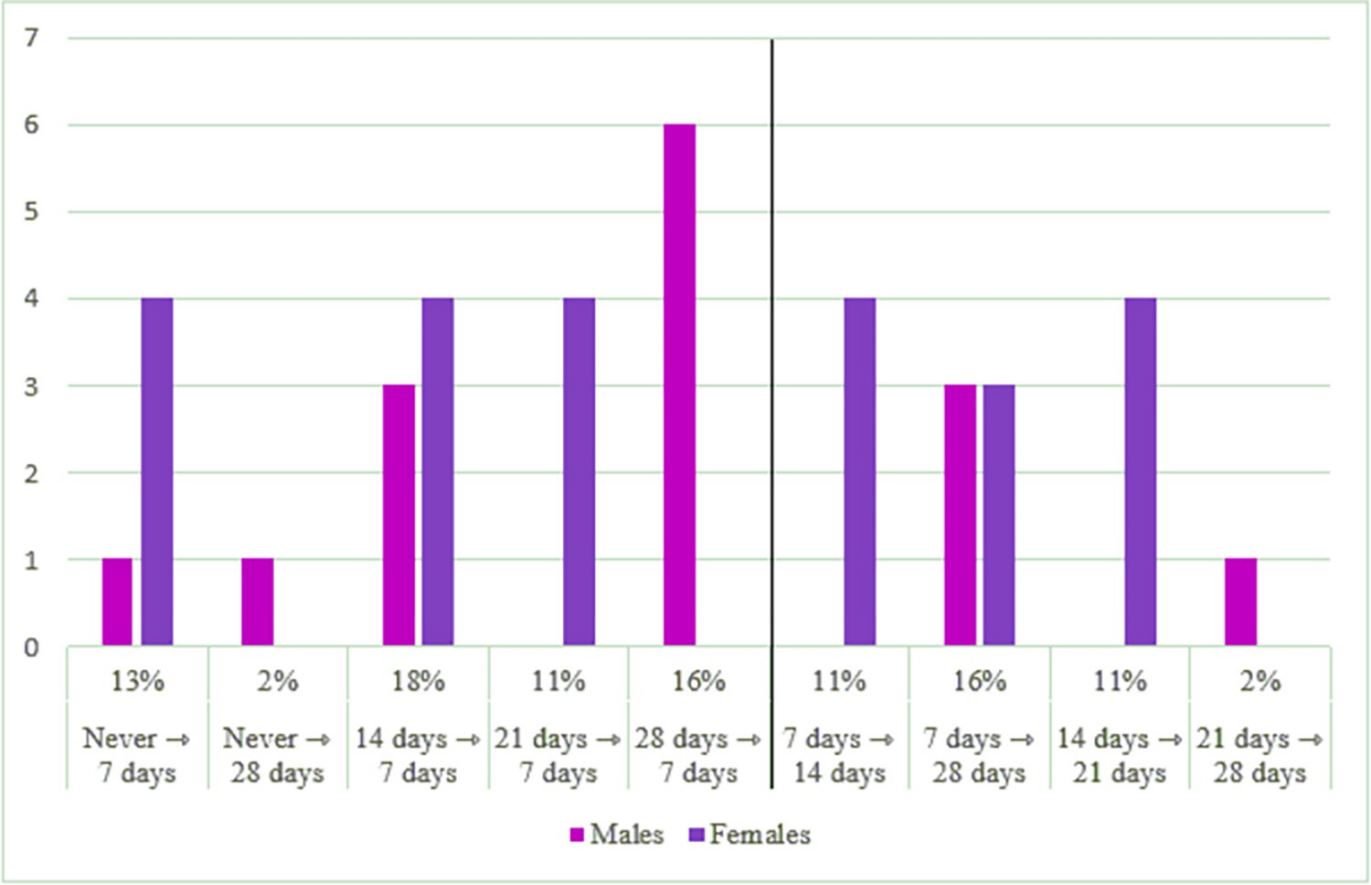
Preference changes for radiology and pathology test results.

Fig 2 shows that 23 patients (60%) preferred to get their test results quicker, seven (18%) of whom changed their preferences from 14 days to 7 days. Furthermore, 15 patients (40%) extended their time delay for getting their test results.

In sum, Fig 1 shows that 47 (78%) changes were made for laboratory results to a shorter delay and 7 (22%) changes to a longer delay. Fig 2 shows that 23 (60%) changes were made for radiology and pathology results to a shorter delay and 15 changes (40%) to a longer delay.

Nine patients (6 males, 3 females, mean age 53.8 years) changed their preferences for both their laboratory and radiology and pathology test results at least once in at least 10 specialties (including surgery, ophthalmology, orthopedics, cardiology, dermatology, gastroenterology, internal medicine, pulmonary medicine, neurology, and obstetrics and gynecology). Table 5 shows how many patients changed their preferences for laboratory, radiology and pathology test results in each specialty category. Some patients changed preferences for both results for several specialties. Specifically, two female patients changed their preferences for both results in four specialties, one male patient changed his preferences for both results in two specialties, and four male patients changed their preferences for both results in one specialty.

**Table 5:** Changes in preferences for laboratory and radiology and pathology results per specialty category.

Specialty category (total choices)	Total changes (% of total choices per specialty category)	Male	Female
Surgical (2538)	6 (0.24)	4	2
Medical (5102)	8 (0.16)	3	5
Obstetrics and gynecology (1049)	1 (0.1)	0	1
Unknown (2738)	2 (0.07)	1	1
*Total*	*16*		

#### Reasons for changing initial preferences

Our quantitative analyses showed that most patients preferred to get their laboratory and/or radiology and pathology test results in the shortest time while a small number preferred to wait longer. We interviewed seven of those participants who changed their preferences to gain more insight into why their preferences changed. First, we describe our participants’ experiences in choosing when to receive their laboratory, radiology and pathology results. Second, we describe how our participants’ preferences changed. Third, we provide reasons for these changing preferences.

#### Making a choice

Most participants did not find it hard to choose when they preferred to receive their laboratory and/or radiology and pathology results and did not need any support in making their choices.

*“I didn’t think much about it [red: choice option] at all.” [R1]*

*“I thought about it for a while […] maybe when I visited the nurse, but I think if I look at things that I don’t understand or want to know more about, then I can ask those questions. Again, a longer time, I didn’t want that. So, it was easy for me to make that choice.” [R2]*

Two participants found the warning before looking at the results helpful in making their decision. One participant said:

*“I think the comment on the portal, ‘note you can see results before you have had a medical consultation’, I think that’s fine.” [R5]*

All participants looked at their laboratory and/or radiology and pathology results alone or together with their partner (n = 2) depending on the situation. One participant declared:

*“I’m just curious, I want to know as soon as possible. And when I’m reading and my partner happens to be around, he says ‘oh let me read it too’.” [R7]*

Our participants said that the option to receive radiology results online after 7 days had little value because of how the examination and consultation were organized. Participants explained they had a medical consultation in the hospital discussing the results of the examination on the same day of the examination. One participant said:

*“If you have had an ultrasound or have had an image [red: X-ray] taken, you usually have an appointment with the doctor right away, so you will hear about it anyway. For that kind of result, it doesn’t really matter whether you have the option to know it right away or in fourteen days, because you always hear it that same day.” [R1]*

Radiology test results are disclosed on the portal as written information. Some participants suggested that the images should be included on the portal to help understand the written results. Participants currently have to ask for their images at the reception desk if they want them. One participant explained:

*“And radiology, usually the text appears later I noticed. You can receive the image [read: X-ray], but you just must ask for it at the desk. They make a printout for you.” [R1]*

However, during the COVID-19 pandemic, results were discussed on the phone instead face-to-face with the doctor. In this scenario, the option to receive radiology test results in the shortest time was useful to the participants because this meant they could look at the results during their medical consultation. One participant described the new situation:

*“Before the COVID-19 pandemic, I had an MRI and an examination in the morning and in the afternoon, I discussed these results with the neurologist and neurosurgeon. But during the pandemic, I had to appear for the examination at the hospital, but I only received the results by phone a few days or a week later. I don’t like that. I hope that face-to-face consultations return [..] On the phone you are a bit overwhelmed by what you hear, good or bad, and then you don’t ask questions so quickly. And if you meet a doctor […] they also show the visual display, and then you sit in front of it and then you keep asking. So, you say ‘oh it’s so big or small or changed’, […] but you don’t ask that on the phone. That is why you want to check those results online.” [R7]*

#### The changes made

Our participants changed their preferences from the shortest time delay to longer time delays and visa versa. One participant changed his preferences more than once after experiencing that a longer time delay was not meaningful. He changed his preferences from as soon as possible to a longer delay and then back to as soon as possible:

*“I thought in the beginning, what’s the point of it [red: 1 day disclosure for laboratory and 7 days for radiology and pathology], but I actually liked it anyway. You can prepare yourself a bit for the conversation with the physician who prescribed the examinations and otherwise […] you are in suspense of the results […] It was simply a wrong choice to change to the longer time delay.” [R1]*

Four participants who had changed their preferences for the laboratory, radiology and pathology results could not remember doing so because it was too long ago. One participant declared:

“*It could be*. *But I don’t know anymore*. *It’s been over a year since I made that choice*.*” [R3]*

Three of these four participants expressed that they would prefer to look at their results as soon as possible, indicating that they changed their initial preferences of a longer time delay to a shorter delay:

*“Well, I actually liked that I could look at the results as soon as possible. I do remember that I could tick that option. Because there was also one option that you could look at your results after you’ve been for a medical consultation. I deliberately did not choose this option.” [R2]*

#### Reasons for the changes

Our participants had various reasons for choosing the shortest time delay for receiving their laboratory and/or radiology and pathology results on the patient portal. The first reason was the transparency of health-related information and communication about and with patients. For example, one participant explained:

*“It’s about you. It’s your file. It’s not that it [red: information] stayed with ‘the white coats’ behind closed doors anymore. You can now look at it [red: your file] yourself. It’s about you and your results. I’m just someone who wants to know what’s going on.” [R7]*

Second, choosing the shortest time delay gave the participants time to cope with bad results at home, before consulting the doctor in the outpatient clinic. One participant said:

*“[…] Imagine if I had read, it [red: residual tumor] has grown considerably, then I’m sure the doctor would have said: ‘we have to irradiate again’. The moment I know I can already process that for myself in my head. […] Subsequently I can mentally cope with that a bit and mentally prepare myself for that again. And if that’s the case, then I can handle it well. Because I can just say to myself, it’s for your health, it’s just a must, it’s not fun and I’m just going to ask for more anesthesia or more this or that and then it will be okay. I’ll have to let that sink in and process it for a while. The sooner I know such a result, the better it is.” [R7]*

Third, looking at test results sooner reassured those patients who were worried about their results. One participant declared:

*“If you wait for your blood tests then you are damned busy with that, that there is nothing bad or whatever. And when you look at the results and see that the results all marked green, yes, that’s a bit reassuring.” [R6]*

Fourth, choosing the shortest time delay for laboratory and/or radiology and pathology results gave participants time to prepare for the medical consultation with their doctor. One participant explained:

*“I like that. They [red: doctors] always look at the same thing, I always like to see how the situation is, so then I know, it is neatly indicated whether those values are too high or too low. Then you actually know it prior to the consultation with the doctor, which is a week afterwards. What will happen and what can I do about it.” [R1]*

Fifth, choosing the shortest time delay allowed patients to monitor their results and act on them. One participant described how fast online access to his results was preventive. For example, he immediately started to lose weight when he found out that his sugar level had risen:

*“I can now compare my results to the last time. And it [red: the portal] also shows between which values my blood results should be. For example, between what value your sugar level should be, whether the result is on average, below or above it. As a non-medical person, I can follow it myself. […] but I know that if I let go of the reins, those values go up. When I see that my values are rising, I know that I must tighten up the regime.” [R3]*

The last reason for choosing the shortest time delay for laboratory and/or radiology and pathology results was to learn more about what the results mean by monitoring and sharing them with the general practitioner. One participant explained that she learned a lot from the values on the patient portal by monitoring the results, asking questions, and taking the results to her general practitioner for more insights:

*“For example, you can study what is changed. There is a graph with it [red: lab results] and you can see if everything is good. But I should also have to do a blood test at the doctor’s office for cholesterol levels and then I saw that on the portal of the hospital that the values deviate very much from one and the other. I just learn a lot from it. At the hospital my values were good and at the general practitioner my values were just way too high. And then I could ask all my questions about that. Subsequently you just know and otherwise you don’t know those kinds of things.” [R2]*

*“I think that’s really great, because I can take the test results, e.g., my cholesterol, to my general practitioner, that’s nice.” [R2]*

Some participants chose a longer time delay for receiving their laboratory and/or radiology results. Two participants changed their preferences from the shortest time to a longer delay (28 or 7 days) for two reasons. First, one participant remembered the first time he looked at the patient portal and felt very disappointed by the lack of profound information on there:

*“At that moment I was very disappointed. You gain access to the portal. You will see some information, where you have appointments and things like that. But there is no profound information. What was discussed with the doctor was not in it [red: the portal]. That is probably contained in a certain note that is not transparent, so to speak. Additionally, what is insightful were my blood results. As time goes by, you also notice that the portal is filled with old information from the past. This information is nice, but no longer relevant. […] at that moment what I wanted to find; I couldn’t find.” [R4]*

Second, participants opted for a longer delay because information on the portal was incomprehensible. It was incomprehensible because the text contained a lot of medical jargon and the lab results were not presented with contextual information, such as what is normal. Two participants tried to understand their results by searching on the internet, but did not find any explanations they could understand. In the end, they stopped doing this because the information they found just made them anxious. One participant illustrated:

*“I concluded ’do I want this?’ […] I remember looking at a value that was marked red […], then I searched on the Internet and I found a story that I couldn’t do anything […] Then I thought, I really shouldn’t search anymore, I just have to trust the doctor, who knows hundred times more about this [red: values] than I do, so I just have to wait for the doctor. I was naive about it, and I thought I have a look and then it [red: all the information] was a lot for me to take in.” [R5]*

Despite changing their initial choice to a longer time delay, these participants valued that their health-related information was transparent and said that they now preferred to see their results directly after the medical consultation with their doctor.

*“Looking back at your values is nice, because you can look for something specific in terms of values, and compare them to how they were before.” [R5]*

## Discussion

We studied the choices patients made about receiving their laboratory and/or radiology and pathology test results on a patient portal and their reasons for changing these initial preferences. Our findings show that most patients preferred the shortest time delay for receiving their laboratory and/or radiology and pathology results (Table 2). We also found that a small number of patients preferred a longer time delay for their results (Table 2) and that some patients changed their initial preferences (N = 43). Interviews with these patients gave more in-depth insights into these initial choices and why their preferences changed. Our participants did not find it hard to make decisions about when they wanted their test results to be disclosed and did not need or want support in making this choice. Moreover, most of our participants looked at their test results alone.

Our finding that most patients want to see their test results as soon as possible is in contrast to the results of Bruno et al [24], who found that most patients prefer a time delay before receiving sensitive test results (such as a diagnosis of Alzheimer’s disease, fetal miscarriage, and cancer) but not for less sensitive test results (such as a diagnosis of high cholesterol, strep throat, genetic disease, and sexually transmitted disease). A possible explanation for the differences between our findings and those of Bruno et al [24] is that Bruno et al [24] asked the participants for their opinion in a survey rather than giving them the option to make the actual choice. In line with our findings, another qualitative study showed that 30 patients with cancer were in favor of real-time disclosure of test results and did not want to wait for their test results because this caused more anxiety than accessing the results [4].

In contrast to our result that patients prefer real-time disclosure of radiology results, Cooper et al [13] showed conflicting findings on patient’s views on the real-time disclosure of radiology results. These differences vary from preferring real-time access or preferring looking at results when the medical consultation took longer than 6 days, or even after 11 days waiting for a telephone call from the doctor [13]. These differences may be explained by differences in timing of the medical consultation. This is in line with our finding that patients prefer real-time disclosure of their radiology and pathology results when their medical consultation with the doctor takes place on the same day of the examination.

We identified various reasons why patients prefer the shortest time delay for disclosure of their test results. First, our patients wanted their health information and communication to be transparent, which is in line with the findings of multiple other studies [2,3,5,7,25]. Second, our patients wanted time to process bad results at home, which was also reported in another study [4]. Third, patients want to see their results quickly for reassurance, which was also found in a systematic review on the impact of patient access to medical files [5]. Fourth, our patients wanted to know their results so they could prepare for their medical consultation, in line with the findings of other studies [1,4,5,9]. Fifth, our patients wanted access to their results so they could monitor and act on them if need be, in agreement with the results of a mixed method study on real-time disclosure of test results for increasing engagement and care utilization of patients with diabetes [9]. Finally, our patients wanted to learn about their results and share them with their general practitioner. This learning aspect is in line with findings of Rexhepi et al [4], but our finding that patients want to share results with their general practitioner is novel to our study.

Based on our findings and those of previous studies, we generally recommend that (hospital) policy makers allow laboratory and radiology results to be disclosed on patient portals as quickly as possible. Although some of our participants lengthened their initial preferences. Remarkable four of these could not remember changing their initial choices, so this may not reflect a true change of preference. Some participants described how emotional concerns, such as disappointment and anxiety due to information on the patient portal being incomprehensible, motivated them to extend their initial choices to a longer time delay. In agreement with this finding, other studies have reported that anxiety and incomprehensibility are negative experiences for patients [5,9,10].

Despite these negative emotional experiences, participants who extended their initial preferences to a longer time delay did appreciate that their health-related information was transparent and accessible. Furthermore, these negative experiences can be avoided by informing and instructing patients and by being transparent upfront about real-time disclosure of test results [7,10]. Besides having information, patients need competences to understand the options of the various choices or need help from the healthcare professionals [26].

The negative experiences of real-time disclosure seem to apply to a small number of patients. The same negative emotions also make healthcare professionals reluctant to disclose test results on the portal in real-time. Healthcare professionals in outpatient clinics are concerned about patient anxiety and health-related information on the patient portal being misunderstood [3,6,9]. Previous research has shown that doctors prefer to give patients their radiology results directly at the consultation rather than disclosing them first on the portal [27], probably to avoid these negative experiences.

Our findings support the importance of comprehensible information on the patient portal, because our participants experienced the patient portal as incomplete and incomprehensible as not all health-related information is accessible. For example, patients prefer to see images instead of just text when accessing their radiology and pathology test results. In agreement with our finding, Cabarrus et al [28] found that 85% of respondents wanted access to their images and radiology reports, even though they were difficult to understand [6,13,29]. A recent study showed that plain language definitions and diagrams helped patients to understand their radiology results [29] and Garry et al [15] have called for further research to evaluate how disclosure of test results affects patient understanding of these results. Crameri et al. [30] have also called for more research on patients’ preferences and needs because patient portals are still not being used to their full potential.

## Conclusions

Our findings show that most patients prefer the shortest possible delay for disclosure of their test results on a patient portal. Reasons for wanting immediate access to test results included a desire for health-related information to be transparent; having time to process bad results at home; reassurance; preparation for medical consultations; monitoring and acting on deviating results to prevent worsening of disease; and learning from the results and sharing them with the general practitioner. A small number of patients preferred a longer delay because they were concerned about the disappointment and anxiety they will feel if the information disclosed on the portal is difficult to understand. In conclusion, our study indicates that most patients want their health-related information to be transparent and disclosed as soon as possible.

## Limitations

Our study has several limitations. First, our qualitative analyses involved a small sample (N = 7, response rate 16%), and more participants are needed to gain more insights into patient preferences. We only interviewed participants who changed their initial preferences (7/43 of in total 4592 participants) to gain more insight into why their preferences changed. We suggest studying especially participants who made an initial choice for a delay of more than 7 days (laboratory results) or more than 14 days (radiology and pathology results), to understand more about their initial choices in a follow-up study. The participants provided us with rich information on their reasoning and helped us to understand how they chose and why they would like to change their initial choice. We did not study their initial choice, which could add more insights. Non-participants provided reasons for not-participating not connected to the aim of our study, e.g., they had no time, no interest in the topic, or no recollection of their choices. However, our findings should be generalized with caution. Future research would add further valuable insights into for example, patients’ opinions on their initial preferences, especially on their reasons for choosing a longer delay. And further research, particularly from other healthcare sectors such as mental healthcare, would probably lead to different results than those reported here, as Van Rijt et al [31] point out at the particular challenges of access to healthcare information in crisis situations. A final limitation is that we only evaluated patient perspectives and not those of the health care professionals.

## Supporting information

S1 Appendix Target group: Patients who changed their initial preferences on laboratory (blood and urine), radiology and pathology test results and on the patient portal. (DOCX)
