# Supplementary material for: Patients’ choices regarding online access to laboratory, radiology and pathology test results on a hospital patient portal
Source: PLoS One. 2023 Feb 3;18(2):e0280768. doi: 10.1371/journal.pone.0280768 (PMC9897579; doi:10.1371/journal.pone.0280768)
Supplement: S1 Appendix — (DOCX) [file pone.0280768.s001.docx]

**S1 Appendix topic list**

Target group: patients who changed their initial preferences on laboratory (blood and urine), radiology and pathology test results and on the patient portal.

| **Open questions about the patient portal and test results** | - How long have you been using the patient portal? - How did you learn about the existence of this portal? - How did you learn about the existence of online access to test results on the portal? - How were you informed about online access to test results through the patient portal? - Informed? 🡪 What do you think of this way of informing? - Not informed? 🡪 How would you like to have been informed about viewing your test results? In what way/by whom? - What do you think of being able to view your research results online through the portal? - How often have you used the portal to view your test results? - What are your experiences with viewing your test results online? - What do you think is an advantage? Can you give an example? - What do you think is a disadvantage? Can you give an example? - What did you do with the information that you read on the portal? - Do you remember the first time you looked at your test results on the portal? Can you tell me how that was? Where you alone or with others? Was that a conscious choice? How did you experience that? - How understandable did you find the information you read? - Understandable? 🡪What did you do with this information? How did you feel about it? - Incomprehensible? 🡪 How did that happen? How did you feel about that? What should be done to make the information understandable for you? What have you done with the information you read on the portal? |
| --- | --- |
| **Open questions about the options of online access** | Since October 2020, you can choose within which timeframe you want to view your test results on the patient portal (1,7,14, 21, and 28 days or no access).  What do you think of being able to choose from different options to view your test results?  How do you feel about being able to choose different options for both laboratory results and radiology/pathology results?   - How were you informed about these options on the portal? - Informed? 🡪 What do you think of this way of informing? - Not informed? 🡪 How would you have liked to be informed about viewing your test results? In what way/by whom? - May I ask what you have entered in terms of delay time for laboratory results? (1, 7, 14, 21, and 28 days or no online access) - What are your main reasons for choosing X number of days of delay? - How did you make this choice? - How did you feel about making this choice? - Did you think about the choice for a long time or not? Can you explain that? - Did you receive help/guidance in making this choice? - Yes? 🡪 In what way/how? How do you look back at the help/guidance? - No 🡪 Did you need help/guidance? And can you tell me what you would have liked? - What do you think of the options for the laboratory results: 1, 7, 14, 21, and 28 days or no access? - Why good? Can you give an example? - Why bad? Can you give an example? - What needs to be changed or improved? - May I ask what you have entered in terms of delay time for laboratory results? (7, 14, 21, and 28 days or no online access) - What are your main reasons for choosing X number of days of delay? - How did you make this choice? - How did you feel about making this choice? - Did you think about the choice for a long time or not? Can you explain that? - Did you receive help/guidance in making this choice? - Yes? 🡪 In which way/how? How do you look back at the help/guidance? - No 🡪 Did you need help/guidance? And can you tell me what you would have liked? - What do you think of the options for the radiology/pathology results: 7, 14, 21, and 28 days or no access? - Why? Can you give an example? - What needs to be changed or improved?   *Possible questions (depending on whether there is a difference in choices)*   - *There is a difference in the choices you have made for the laboratory results and radiology/pathology results. I heard that you want to see your laboratory results [as soon as possible] and in radiology and pathology after [14, 21, 28 or not] days. How did you make this choice? / What is this choice based on? Why is there a difference in the number of days?* - *There is a difference in the choices you have made for the laboratory results and radiology/pathology results. I heard you want to see your radiology and pathology results within 7 days (earliest possible) and your laboratory results after [14, 21, 28 or 0] days.* - *How did you make this choice? / What is this choice based on? Why is there a difference in the number of days?* |
| **Open questions regarding to changing preferences** | You have changed your preferences. Do you want to tell me which preferences you have changed in the portal?   - For laboratory or for radiology/pathology results? - Can you tell me why you changed your choice? - What do you experience as an advantage that you can now view the results after X number of days instead of Y number of days? - Can you give an example? - What do you experience as a disadvantage that you can now view the results after X number of days instead of Y number of days? - Can you give an example? |
